# Patients with preexisting psychiatric disorders admitted to ICU: a descriptive and retrospective cohort study

**DOI:** 10.1186/s13613-016-0221-x

**Published:** 2017-01-03

**Authors:** Arnaud Gacouin, Adel Maamar, Pierre Fillatre, Emmanuelle Sylvestre, Margaux Dolan, Yves Le Tulzo, Jean Marc Tadié

**Affiliations:** 1 Service des Maladies Infectieuses et Réanimation Médicale, Maladies Infectieuses et Réanimation Médicale, CHU Rennes, 35033 Rennes, France; 2 Faculté de Médecine, Biosit, Université Rennes 1, 35043 Rennes, France; 3 Inserm-CIC-1414, Faculté de Médecine, Université Rennes 1, IFR 140, 35033 Rennes, France; 4 Département d’information médicale, CHU Rennes, 35033 Rennes, France; 5 INSERM, U1099, 35000 Rennes, France; 6 LTSI, Université de Rennes 1, 35000 Rennes, France; 7 Département de psychiatrie, Centre Hospitalier Guillaume Regnier, CHU Rennes, 35703 Rennes, France

**Keywords:** Critical care, Cohort study, Self-harm, Psychiatric diagnoses, Psychoactive medication, Outcome

## Abstract

**Background:**

While the psychiatric disorders are conditions frequently encountered in hospitalized patients, there are little or no data regarding the characteristics and short- and long-term outcomes in patients with preexisting psychiatric disorders in ICU. Such assessment may provide the opportunity to determine the respective impact on mortality in the ICU and after ICU discharge with reasons for admission, including modalities of self-harm, of underlying psychiatric disorders and prior psychoactive medications.

**Methods:**

ICU and 1-year survival analysis performed on a retrospective cohort of patients with preexisting psychiatric disorders admitted from 2000 through 2013 in a 21-bed polyvalent ICU in a university hospital.

**Results:**

Among the 1751 patients of the cohort, 1280 (73%) were admitted after deliberate self-harm. Psychiatric diagnoses were: schizophrenia, *n* = 97 (6%); non-schizophrenia psychotic disorder, *n* = 237 (13%); depression disorder, *n* = 1058 (60%), bipolar disorder, *n* = 172 (10%), and anxiety disorder, *n* = 187 (11%). ICU mortality rate was significantly lower in patients admitted after self-harm than in patients admitted for other reasons than self-harm [38/1288 patients (3%) vs. 53/463 patients (11%), respectively, *p* < 0.0001]. Compared with patients admitted for deliberate self-poisoning with psychoactive medications, patients admitted for self-harm by hanging, drowning, jumping from buildings, or corrosive chemicals ingestion had a significantly higher ICU mortality rate. In the ICU, SAPS II score [adjusted odds ratio (OR) 1.061, 95% CI 1.041–1.079, *p* < 0.0001], use of vasopressors (adjusted OR 7.40, 95% CI 2.94–18.51, *p* < 0.001), out-of-hospital cardiac arrest (adjusted OR 14.70, 95% CI 3.86–38.51, *p* < 0.001), and self-harm by hanging, drowning, jumping from buildings, or corrosive chemicals ingestion (adjusted OR 11.49, 95% CI 3.76–35.71, *p* < 0.001) were independently associated with mortality. After ICU discharge SAPS II score [adjusted hazard ratio (HR) 1.023, 95% CI 1.010–1.036, *p* < 0.01], age (adjusted HR 1.030, 95% CI 1.016–1.044, *p* < 0.0001), admission for respiratory failure (adjusted HR 2.23, 95% CI 1.19–4.57, *p* = 0.01), and shock (adjusted HR 3.72, 95% CI 1.97–6.62, *p* < 0.001) were independently associated with long-term mortality. Neither psychiatric diagnoses nor psychoactive medications received before admission to the ICU were independently associated with mortality.

**Conclusions:**

The study provides data on the short- and long-term outcomes of patients with prepsychiatric disorders admitted to the ICU that may guide decisions when considering ICU admission and discharge in these patients.

## Background

Mental disorders are increasingly involved in premature mortality as well as years living with a disability [1]. Although patients with psychiatric diagnoses are common among severely ill patients and may represent a significant proportion of those treated in the ICUs [2, 3], there are no data regarding the epidemiology and impact of psychiatric disorders on ICU outcome. The largest studies assessing the impact of psychiatric comorbidity on outcomes were mostly performed in patients with acute myocardial infarction and reported conflicting results [4–6]. Although there are extensive published data on the risk of subsequently developing psychiatric disorders after an ICU stay [7–10], fewer data on the impact of preexisting psychiatric diagnoses on short- and long-term ICU outcomes are available [3, 4, 11–13].

Mental disorders cover a large number of varied pathologies, inducing specific care with specific therapies. For instance, a previous study has shown that schizophrenic disorders may have a negative impact on outcomes in medical ICU [13] and in a large cohort of non-critically ill patients with preexisting disorders; Thompson et al. [14] have demonstrated that patterns of hospital admission for adult psychiatric illness induced dramatic changes in patients’ outcome.

Thus, nature of mental disorders, reasons for hospital admission, and preexisting medications should be taken into account when studying patients with preexisting mental disorders in ICU. To our knowledge, the respective impact of specific psychiatric disorders and reasons for admission including self-harm and other reasons for admission on short- and long-term outcomes in patients with psychiatric disorders admitted to the ICU has not been previously assessed in a large population. Such a study may help to describe reasons for admission to the ICU and assess risk factors for mortality in critically ill patients with psychiatric disorders in industrialized countries. For that purpose, we performed a retrospective cohort study on patients with various psychiatric disorders admitted in our ICU from 2000 to 2013.

## Methods

### Patients and setting

This retrospective study was performed in a mixed 21-bed ICU admitting mostly medical patients at the Rennes University Hospital, a tertiary teaching hospital. Patients were identified through: (1) the computerized database of the department of intensive care unit (ICU); (2) the hospital discharge database from the Program of Medicalization of the Information System (PMSI) using *the International Classification of Diseases, 9th and 10th Revisions* (ICD-9 and ICD-10). The study was approved by the hospital’s ethics committee (No. 14-95), and the database was declared to the Commission Nationale de l’ Informatique et des Libertés (No. 8274120). We included all medical patients aged over 18 years admitted to the ICU for the first time between January 1, 2000, and December 31, 2013, with at least one of the following psychiatric diagnoses: depressive disorders (ICD 9/10 296.2 and 296.3; 311; 300.4; F32; F33), anxiety disorders (300–300.02; 293.84; 309.28; 309.21–309.23; F40 and F41), bipolar disorders (296.00–296.06; 296.40–296.89; F30; F31), and psychotic disorders including schizophrenia (295; F20–F29). Of note, patients with substance dependence (alcohol and/or drug addiction, ICD 9/10 303 and 304; F10–19) were distinguished from the other patients, and their medical records were carefully reviewed for these diagnoses [15]. Lastly, despite specific differences, underlying psychiatric disorder and self-poisoning sometimes overlap. In the present study, we included only patients with known psychiatric diagnoses. Patients were excluded when deprived of their liberty, and only the first hospitalization was included in the analysis. Follow-up information was obtained from hospital records and when available from the Civil Registration System of the city in which patients were born. The outcome was based on survival at January 1, 2015, which was used as the end of the study period to ensure that all patients had at least 1 year of observation time.

### Data collection and definitions

Data were extracted from medical records through a standardized questionnaire. The baseline data collected for all patients were: age, sex, and the Simplified Acute Physiology Score (SAPS) II calculated within 24 h after admission [16]. Patients were distinguished whether they were admitted after deliberate self-harm or not, and whether they were directly admitted from a psychiatric hospital or psychiatric ward or not. Four groups of self-harm patients were distinguished: (1) deliberate self-poisoning with psychoactive medications alone, (2) deliberate self-poisoning with non-psychoactive medications alone or associated with psychoactive medications, (3) deliberate self-poisoning with pesticides, household, or industrial products, alone or associated with psychoactive medications, and (4) deliberate self-harming by hanging, drowning, jumping from buildings, or corrosive chemicals ingestion alone or associated with psychoactive medications. The following variables recorded upon ICU admission and during the ICU stay were: intubation and invasive mechanical ventilation, renal replacement therapy, vasopressors or inotropic therapy including the use of epinephrine, norepinephrine, and dobutamine, ICU acquired infections at any sites as defined previously [17], and ICU mortality. In addition, chemical pneumonitis and aspiration pneumonia were recorded [18]. Regarding the psychoactive medications received by patients before admission to the ICU, we considered the treatments described in medical records as received regularly by patients during a minimum of 6 months prior to ICU admission. The following psychoactive treatments were recorded: benzodiazepines, neuroleptics, antipsychotics other than neuroleptics, lithium, selective serotonin reuptake inhibitors (SSRIs), serotonin and norepinephrine reuptake inhibitors (SNRIs), tri- and tetracyclics antidepressants, and monoamine oxidase inhibitors (MAOIs). Additionally, cause-specific death was assessed retrospectively, based on chart abstraction. The proximate cause of death was defined as the pathology leading to the death of the patients or the decision to withhold or withdraw intensive care.

### Study end points

End points included assessment of general characteristics of patients, reasons for admission to the ICU, identification of variables independently associated with ICU mortality and 1-year mortality after ICU discharge, and assessment of proximate causes of death.

### Statistical analyses

Data are summarized as number (percentages) for categorical variables. Quantitative variables are expressed as mean ± SD if normally distributed and median (interquartile range) if non-normally distributed. As appropriate, qualitative data were compared using Chi-square test or Fisher exact test while quantitative data were compared using unpaired *t* test or Mann–Whitney test. We used a logistic regression analysis to identify variables independently associated with mortality. Variables with a *P* value ≤0.10 in the univariate analysis were entered in the model, and results were expressed as odds ratios (OR) with their 95% confident interval (CI). A univariate Cox regression was used to identify variables with potential prognostic significance after ICU discharge, and the Cox proportional hazards model was used to assess the independent contribution of each predictor. Variables were removed in a backward stepwise selection process on the basis of a significance level of 0.10 in the univariate analysis. Patients who were alive at the end of follow-up were censored, and results were expressed as hazard ratio (HR) and their 95% (CI). All probability values reported are two-sided. Statistical analysis was performed with the use of Statistical Package for Social Sciences, version 17 (SPSS, IBM, Chicago, IL, USA) and Statview 5 (SAS Institute, Cary, NC, USA) statistical software package.

## Results

### Patients and descriptive data

Between January 1, 2000, and December 31, 2013, 13,715 patients were admitted to the ICU, and 1834 patients (13%) with known psychiatric disorders were eligible for the study. After reviewing medical records, 91 patients were excluded because they had been admitted in the ICU before January 2000 (*n* = 56) or deprived of their liberty (*n* = 35), and finally, 1743 patients were included into the study. Characteristics of patients including diagnosed psychiatric disorders, psychoactive treatments, and reasons for admissions are listed in Table 1. Reasons for admissions were deliberate self-harm in 1280 patients (73%) and other than deliberate self-harm in 463 patients (27%). Among the non-psychoactive medications recorded in the 139 patients with deliberate self-poisoning, paracetamol was involved in 49 patients, beta blockers in 36 patients, calcium channel blockers in 12 patients, insulin in 6 patients, chloroquine in 4 patients, morphinics in 10 patients. Nineteen patients were admitted after hanging, six patients after drowning, two after corrosive chemicals ingestion, one after jumping from building, and two after gun shot. Organophosphate pesticides were involved in eight patients, paraquat in two patients, strychnine in three patients, ethylene glycol in four patients, solvents in three patients, gas in two patients.

**Table 1:** Baseline characteristics of the study population

Variables	Whole population *n* = 1743
Age [years (mean ± SD)]	43 ± 3
Male gender [*n* (%)]	814 (46)
SAPS II score, points (mean ± SD)	34 ± 17
Mental disorder [*n* (%)]	
Schizophrenia	97 (6)
Non-schizophrenia psychiatric disorder	237 (13)
Depression disorder	1058 (60)
Bipolar disorder	172 (10)
Anxiety disorder	187 (11)
Substance dependence (alcohol and/or drug addiction)	492 (28)
Transfer from psychiatric hospital or ward [*n* (%)]	130 (7)
Psychoactive medication before admission [*n* (%)]	
None	197 (11)
Benzodiazepines	1043 (60)
Neuroleptics	497 (28)
Antipsychotics other than neuroleptics	175 (10)
Lithium	81 (5)
SSRIs	420 (24)
SNRIs	34 (2)
Tri- or tetracyclics antidepressants	226 (13)
MAOIs	148 (8)
Admission following deliberate self-harm [*n* (%)]	1280 (73)
Deliberate self-poisoning with psychoactive medications	
Alone	1082 (62)
And/or with non-psychoactive medications	139 (8)
And/or with pesticides, household, or industrial products	28 (1.5)
And/or with hanging, drowning, jumping from buildings, or corrosive chemicals ingestion	31 (1.5)
Admission for another reason than deliberate self-harm	463 (27)
Respiratory failure	165 (9)
Shock	65 (4)
CNS or peripheral disorder	82 (5)
Metabolic disorder	53 (3)
Out-of-hospital cardiac arrest	18 (1)
Acute kidney injury	13 (0.5)
Non-deliberate psychoactive medication overdose	44 (2.5)
Miscellaneous	33 (2)
Chemical pneumonitis and/or aspiration pneumonia [*n* (%)]	479 (27)
Organ support in the ICU [*n* (%)]	
Invasive mechanical ventilation	1206 (69)
Vasopressors	378 (22)
Renal replacement therapy	176 (10)
ICU outcomes [*n* (%)]	
Acquired infection at any site	64 (4)
Mortality	91 (5)

*SAPS* Simplified Acute Physiology Score, *CNS* central nervous system, *SSRIs* selective serotonin reuptake inhibitors, *SNRIs* selective norepinephrine reuptake inhibitors, *MAOIs* monoamine oxidase inhibitors

### Mortality in the ICU

ICU mortality rate was significantly lower in patients admitted after self-harm than in patients admitted for another reason than self-harm [38/1280 patients (3%) vs. 53/463 patients (11%), respectively, *p* < 0.0001], and it was of 2% in the 1221 patients with deliberate self-poisoning with medications only. However, compared with patients admitted for deliberate self-poisoning with psychoactive medications, patients admitted for self-harm by hanging, drowning, jumping from buildings, or corrosive chemicals ingestion had a significantly higher ICU mortality rate. Table 2 shows comparisons between patients who survived and those who did not survive their ICU stay. Among psychiatric diagnoses recorded, only non-schizophrenia psychiatric disorder was associated with mortality in the univariate analysis. After logistic regression analysis, SAPS II score (adjusted OR 1.061, 95% CI 1.041–1.079, *p* < 0.0001), use of vasopressors (adjusted OR 7.40, 95% CI 2.94–18.51, *p* < 0.001), out-of-hospital cardiac arrest (adjusted OR 14.70, 95% CI 3.86–38.51, *p* < 0.001), and self-harm by hanging, drowning, jumping from buildings, or corrosive chemicals ingestion (adjusted OR 11.49, 95% CI 3.76–35.71, *p* < 0.001) were independently associated with mortality. Neither psychiatric diagnoses nor psychoactive medications received before admission to the ICU were independently associated with mortality. Causes of death following self-harm were as follows: respiratory (*n* = 4, 11%), neurological (*n* = 5, 13%), out-of hospital cardiac arrest (*n* = 18, 47%), septic shock (*n* = 1, 3%), cardiogenic shock (*n* = 6, 15%), and fulminans hepatitis (*n* = 4, 11%). Causes of death in psychiatric patients admitted to ICU for other reasons were as follows: respiratory (*n* = 9, 17%), neurological (*n* = 6, 11%), out-of-hospital cardiac arrest (*n* = 13, 24%), septic shock (*n* = 9, 17%), cardiogenic shock (*n* = 5, 9%), and others (*n* = 11, 20%) (*p* = 0.03 after comparison).

**Table 2:** Baseline characteristics and outcomes compared between patients who died in the ICU and patients who survived the ICU stay

Variable	Deceased patients *n* = 91	Surviving patients *n* = 1660	*P* value
Age [years (mean ± SD)]	54 ± 17	45 ± 15	<0.0001
Male gender [*n* (%)]	46 (51)	766 (46)	0.42
SAPS II score, points (mean ± SD)	69 ± 21	36 ± 15	<0.0001
Mental disorder [*n* (%)]			
Schizophrenia	3 (3)	94 (6)	0.48
Non-schizophrenia psychiatric disorder	16 (21)	218 (13)	0.04
Depression disorder	50 (55)	1008 (60)	0.27
Bipolar disorder	12 (13)	160 (10)	0.26
Anxiety disorder	7 (8)	180 (11)	0.48
Substance dependence (alcohol and/or drug addiction)	14 (15)	478 (29)	0.003
Transfer from psychiatric hospital or ward [*n* (%)]	14 (15)	116 (7)	0.003
Psychoactive medication before admission [*n* (%)]			
None	9 (10)	189 (11)	0.66
Benzodiazepines	58 (64)	1138 (68)	0.33
Neuroleptics	26 (28)	471 (28)	0.97
Antipsychotics other than neuroleptics	10 (11)	165 (10)	0.74
Lithium	3 (3)	78 (5)	0.53
SSRIs	18 (20)	402 (24)	0.33
SNRIs	1 (1)	33 (2)	0.55
Tri- or tetracyclics antidepressants	11 (12)	215 (13)	0.81
MAOIs	8 (9)	140 (8)	0.90
Reason for admission [*n* (%)]			
Deliberate self-poisoning with psychoactive medications			
Alone	15 (16)	1067 (64)	<0.0001
And/or with non-psychoactive medications	10 (11)	129 (8)	0.27
And/or with pesticides, household, or industrial products	0 (0)	28 (2)	0.40
And/or with hanging, drowning, jumping from buildings, or corrosive chemicals ingestion	13 (14)	18 (1)	<0.0001
Respiratory failure	14 (15)	151 (9)	0.06
Shock	15 (16)	50 (3)	<0.0001
CNS or peripheral disorder	6 (7)	76 (6)	0.31
Metabolic disorder	1 (1)	52 (3)	0.52
Out-of-hospital cardiac arrest	14 (15)	4 (0.2)	<0.0001
Acute kidney injury	0 (0)	13 (0.5)	0.99
Non-deliberate psychoactive medication overdose	2 (2)	42 (2.5)	0.99
Miscellaneous	3 (3)	30 (2)	0.24
Chemical pneumonitis and/or aspiration pneumonia [*n* (%)]	28 (31)	451 (27)	0.45
Organ support in the ICU [*n* (%)]			
Invasive mechanical ventilation	91 (100)	1115 (67)	<0.0001
Vasopressors	84 (92)	294 (18)	<0.0001
Renal replacement therapy	38 (42)	138 (8)	<0.0001
Acquired infection at any site	14 (15)	50 (3)	<0.0001

*SAPS* Simplified Acute Physiology Score, *CNS* central nervous system, *SSRIs* selective serotonin reuptake inhibitors, *SNRIs* selective norepinephrine reuptake inhibitors, *MAOIs* monoamine oxidase inhibitors

### One-year mortality after ICU discharge

One hundred thirty-five patients (8%) were lost of view within the year following ICU discharge. Results for unadjusted and adjusted 1-year survival analysis are shown in Table 3. After adjustment, neither psychiatric diagnoses nor modalities used for self-harm remained independently associated with survival (Table 3). In addition to age and SAPS II score, admission to the ICU for respiratory failure or shock was the variables independently associated with prognosis.

**Table 3:** Unadjusted and adjusted 4-year prognostic analysis for the 1660 patients who survived the ICU stay

Variable	Non adjusted hazard ratio	95% Confident interval	*P* value	Adjusted hazard ratio	95% Confident interval	*P* value
Age (1-year increment)	1.053	1.041–1.066	<0.0001	1.030	1.016–1.044	<0.0001
Male gender	1.01	0.69–1.46	0.96			
SAPS II score (1-point increment)	1.037	1.035–1.049	<0.0001	1.023	1.010–1.036	<0.01
Mental disorder						
Anxiety disorder	0.47	0.21–1.07	0.07			
Schizophrenia	1.67	0.87–3.20	0.12			
Psychotic disorder other than schizophrenia	1.57	0.98–2.52	0.06			
Depression disorder	0.80	0.55–1.16	0.24			
Bipolar disorder	1.18	0.65–2.14	0.59			
Substance dependence (alcohol or drug addiction)	1.12	0.79–1.16	0.42			
Transfer from psychiatric hospital or ward	1.79	1.01–3.19	0.04			
Psychoactive medication before admission						
None	1.17	0.72–2.05	0.58			
Benzodiazepines	0.97	0.80–1.90	0.80			
Neuroleptics	1.16	0.78–1.72	0.47			
Antipsychotics other than neuroleptics	1.08	0.80–1.47	0.60			
Lithium	1.59	0.77–3.26	0.21			
SSRIs	0.84	0.53–1.32	0.44			
SNRIs	0.34	0.11–1.05	0.48			
Tri- or tetracyclics antidepressants	0.52	0.21–1.27	0.52			
MAOIs	0.89	0.63–1.24	0.42			
Reason for admission						
Deliberate self-poisoning with psychoactive medications alone	0.30	0.20–0.44	<0.0001			
Deliberate self-poisoning with psychoactive medications and/or non-psychoactive medications	0.11	0.10–0.78	0.03			
Deliberate self-poisoning with psychoactive medications and/or with pesticides, household or industrial products	0.45	0.17–1.19	0.11			
Deliberate self-poisoning with psychoactive medications and/or with hanging, drowning, jumping from buildings or corrosive chemicals ingestion	1.06	0.12–2.84	0.86			
Respiratory failure	3.28	2.11–3.99	<0.0001	2.33	1.19–4.57	0.01
Shock	6.30	3.76–5.92	<0.0001	3.72	1.97–6.62	<0.001
CNS or peripheral disorder	2.18	1.14–4.17	0.02			
Metabolic disorder	1.50	0.61–3.67	<0.38			
Out-of-hospital cardiac arrest	4.52	0.63–7.79	0.13			
Acute kidney injury	4.35	1.27–6.24	0.01			
Non-deliberate psychoactive medication overdose	1.34	0.56–3.71	0.54			
Miscellaneous	1.50	0.48–4.43	0.48			
Chemical pneumonitis and/or aspiration pneumonia [*n* (%)]	1.18	0.67–1.57	0.99			
Organ support in the ICU						
Invasive mechanical ventilation	0.84	0.57–1.24	0.38			
Vasopressors	2.01	1.39–3.10	<0.0001			
Renal replacement therapy	1.89	1.11–2.21	0.02			
ICU outcomes						
Acquired infection at any site	2.62	1.87–5.37	<0.01			

*SAPS* simplified acute physiologic score, *CNS* central nervous system, *SSRIs* selective serotonin reuptake inhibitors, *SNRIs* selective norepinephrine reuptake inhibitors, *MAOIs* monoamine oxidase inhibitors

## Discussion

We performed a retrospective study on a large cohort of patients with five psychiatric diagnoses admitted to the ICU (i.e., depressive, anxiety, bipolar, and psychotic disorders including schizophrenia). Over a 14-year period, this study population represents one in ten patients admitted to our ICU over the study period. Depression disorder was the most frequent psychiatric disorder encountered, and substance dependence was noted in 28% of these patients. Seventy-five percent of patients were admitted after self-harm.

In our study, we found that admissions after self-harm had a lower mortality rate than admissions for other reasons. Although not previously published, these results were expected since patients hospitalized for self-poisoning are young and often used central nervous system acting drugs for self-poisoning that required intubation during few hours only to protect their airways and ensure their ventilation until recovery [2], as found in our study: Patients admitted after self-harm were significantly younger than the other patients (42 years ± 14 vs. 55 years ± 15, respectively, *p* < 0.0001), more frequently women (55 vs. 49%, respectively, *p* = 0.01), and most often used central nervous system acting drugs. Of note, deliberate self-harm with hanging, drowning, jumping from buildings, or corrosive chemicals ingestion whether or not in association with self-poisoning with psychoactive medications was found independently associated with ICU mortality. This finding highlights that ICU outcome mainly depends on physiologic alterations responsible for ICU admission. Along these lines, proximate causes of death in the ICU differed between the two groups of patients. These characteristics could demonstrate that, among patients admitted to the ICU with previously known psychiatric disorders, reason for admission should be taken into account when considering the real impact of psychiatric disorders on ICU mortality.

No study has previously demonstrated that preexisting mental disorders were associated with increased mortality in ICU. In many diseases such as asthma [19] or chronic obstructive pulmonary disease [20, 21], control of the disease and prognostic are poorer in patients with psychiatric comorbidity. These patients could represent a large number of patients admitted to ICU. For instance, in our study, among the 165 patients with respiratory failure as main reason for ICU admission, 78 patients (47%) had a history of chronic obstructive pulmonary disease according to the criteria of the American Thoracic Society published in 1995 [14] and 11 patients (7%) had a history of obesity hypoventilation syndrome. Based on large retrospective studies, Abrams et al. [4] reported that associations with the risk of mortality varied among the individual psychiatric conditions, depression being the psychiatric disease associated with the highest mortality compared to anxiety, psychosis, bipolar disorder, and posttraumatic stress disorder. For other authors [6, 22] and mainly in patients with acute myocardial infarction, the presence of comorbid psychiatric condition is associated with increased short-term mortality. The poorer outcome noted here in patients admitted for another reason than self-harm may be explained in some of them by poor adherence to treatments required by underlying medical condition, admission to the ICU in late stage of their illness because of difficulties in diagnosis [23, 24], or management difficulties during the ICU stay [25]. We hypothesize that psychiatric disorders and/or associated psychoactive medications could lessen symptoms leading to a delayed research on the somatic disease and finally to admission to the ICU patients with advanced forms of their disease.

Lastly, chronic psychiatric medication or associated substance abuse could induce immunosuppression as suggested in several studies and could worsen organ dysfunction and increased risk of nosocomial infections during ICU stay [14]. Along these lines, immunological effects of medications have been evaluated with conflicting results. For instance, it has been shown that both haloperidol and quetiapine significantly increased the level of IL-4, IL-10 and significantly reduced interferon (IFN)-γ production [26] and in vitro studies on antidepressants showed that some antidepressants, such as clomipramine and fluoxetine, more consistently decrease pro-inflammatory cytokines [interleukin (IL)-6, IFN-γ, tumor necrosis factor (TNF)-α]. Furthermore, the impact of mood-stabilizing drugs on cytokine levels remained unclear [27–29].

Our study allowed examination of the effect of psychoactive medications received before admission on short- and long-term mortalities, a variable rarely assessed despite potential prognostic impact [30]. None of the psychoactive medication assessed here and received prior to admission to the ICU was found to have a significant impact on ICU and long-term outcomes. Unfortunately, we were unable to determine whether in the months after discharge psychoactive medication use was increased in patients who received MV, as previously reported by Wunsch et al. [3].

In addition to the retrospective nature of the study, some limitations should be addressed. First, we can’t exclude uncontrolled confounders or case-mix. Second, this is a single-center study, thereby limiting its external validity. Third, post-ICU discharge survival could not be completed in all patients, which may have influenced results, although less than 10% of patients were lost of view during the year following ICU discharge. Fourth, management of patients after ICU discharge was not assessed. Fifth, some may have concern regarding the psychiatric diagnoses in critically ill patients [31]. Abrams et al. [4] showed that the number of patients identified with psychiatric disorders differ whether inpatient psychiatric diagnosis codes or outpatients codes are used. In the present study psychiatric diagnoses were based on in- and outpatient data. Patients with bipolar disorder, schizophrenic and non-schizophrenic psychotic disorders had a specialty-confirmed psychiatric diagnosis. On the other side, we suspect that many patients were diagnosed and treated for depressive or anxiety disorders by general practitioners even if patients with a history of suicide attempts were evaluated by a psychiatrist before hospital discharge. The fact that patients with mental illnesses are frequently diagnosed without referral to a psychiatrist is largely documented [3, 32]. Patients with a depressive disorder could be overrepresented, while those with an anxiety disorder underrepresented in the present study.

## Conclusions

Despite these limitations, we believe that the results of the studies provide data on short- and long-term outcomes in critically ill patients with preexisting psychiatric disorders. In addition, our results suggest distinguishing patients admitted to the ICU after deliberate self-poisoning from the other patients in studies assessing outcomes of critically ill patients with preexisting psychiatric disorders. However, none of the psychiatric disorders were found independently associated with outcomes, even if results of univariate analysis suggest that patients with non-schizophrenic disorders and those transferred from psychiatric hospital or ward may be at particular high risk of poor outcomes. Further studies are needed to confirm that preexisting mental disorders are associated with poorer outcome in patients admitted to ICU.
